# Tin and Gold as a Filling

**Published:** 1888-01

**Authors:** E. G. Betty

**Affiliations:** Cincinnati


### Tin and Gold as a Filling.

BY E. G. BETTY, D.D.S., CINCINNATI.

[Read before the Ohio State Dental Society, Springfield, Ohio, October, 1887.]

So far as the preparation of the cavity is concerned it is done in precisely the same manner as it is for a gold filling, care being taken to have the borders heavy and solid enough to withstand the necessary malleting. The next thing is to prepare the foils for their introduction into the cavity. This may be done in one of several ways. Generally the form most used is the rope, it being made by placing a sheet of No. 4 tin upon a simillar one of non-cohesive gold foil. This is cut into three parts and loosely rolled together. The lengths may be made to suit the size of the cavity to be filled or the tastes of the operator. As a rule they ought not to be so large as to choke the cavity, thereby cutting off vision and impeding rapidity of execution. Probably the best way to start the filling is after the manner in which soft foil cylinders are introduced, placing a piece at each angle of the cervical border and the third one upon these. This is to be continued until the cavity is half or two-thirds full when it may be thoroughly malletted into place. Upon this foundation the gold is built just as though the filling was being started. The finishing is done after the usual methods with file, tape, disk, etc. Some advocate the piling upon each other of a number of alternate sheets of tin and gold and then lightly pressing them together so that they will make a sheet corresponding to No. 80 though somewhat thicker. This is then cut into squares to suit. The pieces are introduced one after another just as cylinders are. If neither of these ways of preparing the foil suits the operator they may be made into cylinders the only requisite being that the tin and gold alternate. In preparing them it does not make any difference which metal is on the outside as far as the integrity of the mass is concerned. It is better, however, to have the gold externally for the reason that it is easier to make the cohesive gold cohere than to the tin. The are several advantages which this combination posesses, one or two of which I may enumerate: It is easy of introduction and in consequence saves a great deal of time to say nothing of being cheaper to the patient than gold. For these two reasons alone it commends itself over amalgam being superior in every respect. In large masses it offers a much better resistence to thermal changes than either gold or amalgam, this point often being very desirable especially when there is great sensitiveness of the dentine. So far as the therapeutical effects of the combination are concerned I have but little to say for the reason that I know nothing further than that a hardening takes place which is accounted for by the action of the buccal fluids upon the two metals, they both being in contact and equally exposed to their action. Whether this is electrical or not I am not prepared to say.

